# Determination of Various Drugs of Abuse in Oral Fluid by a Fabric Phase Sorptive Extraction–LC-MS/MS Method

**DOI:** 10.3390/jox16030077

**Published:** 2026-05-03

**Authors:** Dimitra Florou, Thalia Vlachou, Amvrosios Orfanidis, Vasilios Sakkas, Vassiliki A. Boumba

**Affiliations:** 1 Department of Forensic Medicine & Toxicology, Faculty of Medicine, School of Health Sciences, University of Ioannina, University Campus, 45500 Ioannina, Greece; dm.florou@uoi.gr (D.F.); aorfanidis@uoi.gr (A.O.); 2 Department of Chemistry, School of Sciences, University of Ioannina, 45500 Ioannina, Greece; vsakkas@uoi.gr

**Keywords:** forensic toxicology, oral fluid, drugs of abuse, fabric phase sorptive extraction, LC-MS/MS, validation

## Abstract

Toxicological testing for drugs of abuse (DOAs) is an essential tool for healthcare practitioners and law enforcement agencies. Oral fluid (OF) is an alternative biological fluid for detecting recent DOA intake and is widely employed in forensic investigations. In the current study, a relatively novel and “green” fabric phase sorptive extraction (FPSE) procedure for sample preparation was coupled to liquid chromatography–tandem mass spectrometry (LC–MS/MS) to provide simplicity, cost-effectiveness, rapidity, low solvent consumption, and high analytical performance for the quantitative determination of ten commonly encountered DOAs and metabolites: amphetamine, benzoylecgonine, cocaine, codeine, ecgonine methyl ester, methadone, methamphetamine, 3,4-methylenedioxyamphetamine, 6-monoacetylmorphine, and morphine. The FPSE procedure was optimized by testing different filters, pH, extraction time, and solvents. The validated method demonstrated excellent linearity for all analytes, selectivity, acceptable precision, and high sensitivity (ranges for limits of detection (LODs) and quantification (LOQs) were 0.01–2 ng/mL and 0.03–6 ng/mL, respectively). Autosampler and short-term freeze stability exceeded 95% and 90% for all analytes, respectively. Overall, the combination of FPSE with LC–MS/MS provided a sensitive, selective, and environmentally friendly innovative analytical approach for the determination of DOA in OF and is suitable for both screening and confirmatory forensic and clinical applications.

## 1. Introduction

The phenomenon of substance abuse poses a major threat to society and public health, contributing to significant levels of morbidity and mortality. The worldwide availability of illegal drugs affects people both directly, through use, and indirectly, through the effects on families and communities. Among the various biological specimens used for toxicological analysis and public health monitoring of drugs of abuse (DOAs) in the community, oral fluid (OF) is considered to be the most appropriate non-conventional specimen to assess recent use [1]. OF is slightly acidic (pH 6.2–7.6) and is secreted by the salivary glands into the oral cavity [2,3]. Due to its pH being slightly lower than that of blood, weak basic compounds, such as many DOAs, will be present in OF in higher concentrations compared to blood [1,4]. Furthermore, OF is easily collected with almost no risk of sample adulteration or substitution, enhancing the reliability of analytical results [5].

Fabric Phase Sorptive Extraction (FPSE) is an environmentally friendly, sensitive and effective method of extracting a broad range of analytes from various substrates [6]. FPSE incorporates the advantages of classical extraction procedures such as solid-phase extraction (SPE) and solid-phase microextraction (SPME) by using natural (or synthetic) fibrous substrates covered with hybrid organic–inorganic sol–gel coatings [6,7,8]. Compared to conventional extraction techniques, FPSE offers reduced solvent consumption and simplified workflows, aligning with current trends in green analytical chemistry [6,7,8,9]. However, its application in forensic toxicology remains limited, with previous studies mainly focusing on a small number of analytes or specific classes of novel psychoactive substances [9,10]. On the other hand, the determination of various DOAs in OF based on the liquid chromatography–tandem mass spectrometry (LC-MS/MS) method is widely applied and reported in the literature [1,11,12,13,14,15].

The current contribution reports an incremental extension of the FPSE-LC-MS/MS approach from a single analyte [9] to a multi-analyte OF panel of common DOAs and metabolites, enabling reliable detection of low-level drug exposure and recent drug use to fulfill and substantially surpass current regulatory sensitivity requirements, while also demonstrating promising performance for clinical and forensic applications.

## 2. Experimental Section

### 2.1. Chemicals and Reagents

Analytical standards and compounds were purchased as follows: 6-MAM, Codeine, Morphine, 3,4 methylene-dioxy-amphetamine (MDA), Ecgonine methyl ester (EME): LGC Standards Ltd. (Luckenwalde, Germany). Methadone, amphetamine, methamphetamine, cocaine, benzoylecgonine (ΒΕ): Cayman Chemical Company. MDMA-d5: LGC GmbH (Luckenwalde, Germany). Morphine-d3: Cerilliant Corporation (Round Rock, TX, USA). Ammonium acetate (99%) was purchased from Fluka™ Analytical Standards (Steinheim, Germany) and Dichloromethane was purchased from Fisher Scientific (part of ThermoFisher Scientific) (Waltham, MA, USA). Acetonitrile, methanol, water (all UHPLC-MS grade), and formic acid (99%) were obtained from CARLO ERBA Reagents GmbH (Cornaredo, Italy). Ultra-pure water was supplied by an Aquatron Water Still A4000D purification system from Bibby Sterilin Limited (Staffordshire, UK). Whatman microfiber glass filters (110 mm) and Whatman cellulose filter papers (125 mm), which were used for FPSE, were purchased from General Electric (Boston, MA, USA). The organic polymer polyethylene glycol (PEG 300) came from Sigma-Aldrich (Athens, Greece). Trimethoxymethylsilane (MTMS), trifluoroacetic acid (TFA), acetone, sodium hydroxide (NaOH), and hydrochloric acid (HCl) were supplied by Merck (Darmstadt, Germany). High-performance liquid chromatography (HPLC) grade requirements were fulfilled for all solvents and reagents utilized for LC–MS/MS analyses.

### 2.2. Standard and Working Solutions

Individual stock solutions of all ten analytes were prepared in methanol at a concentration of 1 mg/mL; working solutions were prepared at 100, 10, and 1 μg/mL by dilution with methanol. The IS working solutions were prepared in methanol at a concentration of 10 μg/mL. All solutions were kept at −20 °C until analysis. A pooled drug-free sample of OF (as confirmed by gas chromatography–mass spectrometry) was spiked accordingly to prepare the calibration standards (concentration levels for each substance are shown in 2.9). The lowest calibration level for each analyte was set at or above the corresponding LOQ. Quality control samples were prepared at 6, 20, and 60 ng/mL in methanol.

### 2.3. Pre-Treatment of Fabric for Sol–Gel Coatings

The preparation of Whatman cellulose and microfiber glass filters, including sequential soaking in ultra-pure water, NaOH, and HCl, followed by drying and sol–gel coating with PEG 300/MTMS/TFA catalyst, as well as subsequent rinsing, cutting, and storage, was carried out as described in our previously published work [9].

### 2.4. Preliminary Optimization Experiments

Preliminary experiments were conducted to optimize the FPSE procedure by testing two types of Whatman filters (microfiber glass filter (FG) and cellulose filter (WC)) at three different extraction times—10, 20, and 30 min—and at acidic (5), neutral (7), and alkaline (9 and 12) pH. The tested back-extraction solvents were methanol, acetonitrile, and their mixture of 50:50 *v*/*v*. The back-extraction time was tested in three different time intervals (5, 10, and 15 min). The elution of analytes was tested by sonication and vortexing. Each parameter was tested individually while keeping all other conditions constant.

### 2.5. Sample Collection and Storage

OF was collected from five individual donors and confirmed to be drug-free by GC–MS analysis using a simple expectoration method. OF was allowed to pool in the lower part of the oral cavity, and participants expelled it into a test tube (pre-weighed) at 60 s intervals. Then, OF was vortexed for 30 s and sonicated for 60 s, and 10 mL aliquots were stored in plastic tubes at 5 °C until analysis, with a maximum storage period of two weeks.

### 2.6. FPSE Procedure

First, 100 μL of acetonitrile was added to 1 mL of OF for protein precipitation and then vortexed for 30 s, sonicated for 60 s, and centrifuged at 15,000 rpm for 15 min. The supernatant was subjected to extraction using the selected optimal coated filter for a predetermined time under stirring at 300 rpm and the optimized pH, followed by desorption using 150 μL back-extraction solvent.

### 2.7. LC Conditions

A Dionex UHPLC system (Thermo Scientific, Waltham, MA, USA) coupled to a Q-Trap 5500+™ mass spectrometer (Sciex, Darmstadt, Germany) equipped with an electrospray ionization (ESI) Turbo V source operating in positive mode and in multiple reaction monitoring (MRM) mode was used for chromatographic separation on an Accupore C18 analytical column (50 mm × 3 mm, 2.6 μm) equipped with a precolumn cartridge (2.1 mm × 0.2 μm) (Thermo Scientific, MA, USA), both operated at 30 °C. The injection volume was 5 μL; before and after each injection, the injector’s needle was rinsed with 200 μL methanol. The autosampler temperature was 5 °C. Mobile phases consisted of 10 mM ammonium acetate (pH 3.5, adjusted with 0.1% formic acid) (eluent A) and 0.1% formic acid/acetonitrile UHPLC-MS grade (eluent B). The gradient program at a flow rate of 0.5 mL/min was as follows: 0–0.1 min 2% eluent B; 0.1–0.25 min to 14% eluent B; 0.25–2.5 from 14% to 45% eluent B; 2.5–3.5 from 45% to 100% eluent B; 3.5–4.5 min maintained at 100%; 5.00 min back to initial conditions. The total run time was 6.5 min.

### 2.8. MS/MS Conditions

ESI inlet conditions were as follows: gas 1, nitrogen (55 psi); gas 2, nitrogen (55 psi); nitrogen as the curtain gas at 20 psi; ion-spray voltage of 5500 V, positive mode; ion-source temperature of 550 °C. The scheduled MRM algorithm in Sciex Analyst^®^ software (v. 1.7.1, Sciex, Darmstadt, Germany) under automatic quantitative optimization mode was applied for the optimization of dwell times and other acquisition settings, while data processing was carried out using SciexOS software (v. 1.6, Sciex, Darmstadt, Germany). Table 1 demonstrates the selected MS parameters.

### 2.9. Method Validation

The guidelines of the American Academy of Forensic Sciences were followed for method validation [16].

#### 2.9.1. Selectivity, Carryover, Sensitivity, and Linearity

Selectivity experiments were conducted as follows: Blank OF samples were extracted without the addition of IS (morphine-d3), and with IS. Additionally, blank OF samples were fortified with the following 39 substances (50 ng/mL) to exclude any external interference: agomelatine, alprazolam, a-hydroxyalprazolam, amitriptyline, amoxapine, bromazepam, bupropion, chlordiazepoxide, citalopram, clobazam, clomipramine, desipramine, desmethylcitalopram, diazepam, duloxetine, flunitrazepam, fluoxetine, fluvoxamine, imipramine, lorazepam, maprotiline, midazolam, mianserin, mirtazapine, n-desmethylflunitrazepam, n-desmethyltrimipramine, nitrazepam, nordazepam, nortriptyline, o-desmethylvenlafaxine, oxazepam, paroxetine, prazepam, protriptyline, sertraline, temazepam, triazolam, trimipramine, and venlafaxine.

Blank OF samples were analyzed immediately after the analysis of triplicate high-concentration samples (100 ng/mL) to evaluate potential carryover (signal-to-noise ratio exceeding 3:1 was regarded as evidence of carryover for the respective analytes).

Sensitivity was expressed by the LOD and LOQ, defined as the lowest concentration giving a signal-to-noise (S/N) ratio of three and ten, respectively (determined by analyzing three fortified OF samples at decreasing concentrations from 10 to 0.001 ng/mL).

Five replicates at different concentration levels of the ten substances spiked in the blank OF were applied to test linearity. For each analyte, the lowest calibration point was selected to be equal to the corresponding LOQ. For BE, cocaine and methadone, the calibration concentrations were 0.03, 1, 4, 8, 20, 30, 40, 50, and 60 ng/mL. For amphetamine and methamphetamine, the concentrations were 1, 4, 6, 8, 20, 30, 40, 50, and 60 ng/mL. For 6-MAM and codeine, the concentrations were 3, 6, 8, 20, 30, 40, 50, and 60 ng/mL. For EME and morphine, the concentrations were 4, 6, 8, 20, 30, 40, 50, and 60 ng/mL. For MDA, the concentrations were 6, 8, 20, 30, 40, 50, and 60 ng/mL.

#### 2.9.2. Matrix Effect, Accuracy, Precision, and Stability

Matrix effects and extraction efficiency estimations were performed using the post-extraction addition method at three concentrations, low, medium, and high-quality control (LQC/MQC/HQC), in five replicates each group [17]. Group 1 consisted of neat standards, Group 2 of pooled blank OF samples spiked post-extraction, and Group 3 of pooled OF samples spiked prior to extraction. Matrix effects were assessed by comparing Group 2 with Group 1 at the LQC and HQC (10 x LOQ) levels. The evaluated LQC/HQC concentrations were 1/10 ng/mL for amphetamine and methamphetamine; 0.03/30 ng/mL for cocaine, methadone, and BE; 4/40 ng/mL for EME and morphine; 3/30 ng/mL for codeine and 6-MAM; and 6/60 ng/mL for MDA.

Accuracy and precision experiments were conducted over five consecutive days with three replicates of each low, med (5 × LOQ), and high concentration (LQC/MQC/HQC, respectively), spiked daily with corresponding calibrants as follows: the evaluated LQC/MQC/HQC concentrations were 1/5/10 ng/mL for amphetamine and methamphetamine; 0.03/3/30 ng/mL for cocaine, methadone and BE; 4/20/40 ng/mL for EME and morphine; 3/15/30 ng/mL for codeine and 6-MAM; and 6/30/60 ng/mL for MDA. The accuracy of the method was evaluated as percentage recovery, defined as the percentage difference between the mean of all calculated concentration values at each level and the respective nominal concentration.

Intra-day precision was evaluated using five repetitions daily, while inter-day precision was assessed over five consecutive days. Precision (coefficient of variation %CV) had an acceptable cutoff of ≤20% at all concentration levels.

LQC and HQC samples were used to assess short-term and long-term stability at −20 °C, and on autosampler at 5 °C. Samples were analyzed in triplicate after 24 h (short-term stability) and after one week (long-term stability).

## 3. Results

### 3.1. Method Development

**Figure 1:**
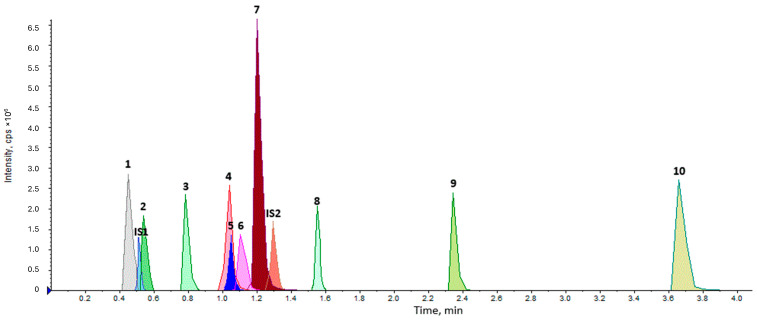
Total Ion Chromatograph (TIC) of the target compounds (1) EME, (2) Morphine, (3) Codeine, (4) Amphetamine, (5) 6-MAM, (6) MDA, (7) Methamphetamine, (8) BE, (9) Cocaine and (10) Methadone at a concentration of 50 ng/mL in OF and the internal standards (IS1) Morphine-d3 and (IS2) MDMA-d5 at a concentration of 50 ng/mL, as well.

Analyte chromatographic separation and MS performance were optimized by evaluating the peak area, peak symmetry, signal-to-noise ratio (S/N), and resolution of respective chromatograms obtained under various tested conditions. An indicative chromatogram of a blank OF spiked with all analytes and ISs is shown in Figure 1.

The FPSE procedure was optimized by evaluating the influence of experimental parameters on analyte recovery. In general, differences among the tested conditions were not statistically significant (*p* > 0.05); therefore, the parameter providing the highest overall performance was selected for further application. The desorption yields obtained with a cellulose microfiber filter (FG) coated with PEG300 were slightly greater than those with fiber glass (except for cocaine) (Figure S1) due to PEG300’s hydrophilic properties and water solubility. An extraction time of 30 min was found to be the most effective for eight out of ten analytes; the exceptions were cocaine and EME, for which the selected time was comparable to 20 min (Figure S2). Neutral pH provided optimal results for most compounds; for amphetamine, this was comparable to pH 9, and for EME, the optimal pH was 9 (Figure S3). Methanol proved to be the most efficient eluent compared to acetonitrile and their mixture, while for codeine, acetonitrile was slightly better, and for EME, the mixture of solvents applied was better (Figure S4). A back-extraction time of 10 min was selected as working better for most analytes (Figure S5). Additionally, the elution of analytes by either ultrasound or vortex showed no significant differences (Figure S6).

### 3.2. Method Validation Results

The validation parameters are summarized in Table 2. The method was found to be selective for the compounds of interest with no endogenous interferences or interferences from co-eluting compounds, including those run as part of the method or from the additional 39 analytes evaluated. Linearity was excellent for all analytes, since all analytes produced R^2^ > 0.990 when using five replicates of seven- to nine-point calibration curves and linear through-zero fit. The ranges of LODs and LOQs were 0.01 ng/mL to 2 ng/mL and 0.03 to 6 ng/mL, respectively. Selectivity experiments revealed no matrix interferences or carryover between runs. Matrix effects were acceptable (response < ±30%) for all analytes. Ion suppression was observed for all analytes except methadone, which showed ion enhancement at low and high concentrations. Intra-day and inter-day precision showed acceptable variability at all tested concentration levels. Accuracy (expressed as recovery %) showed consistent results across low, medium, and high concentrations for most analytes. Only two drugs (EME and morphine) had average recoveries < 15%; nevertheless, these compounds had acceptable responses at the LQC sample analysis (S/N > 10). All analytes showed adequate scores in the autosampler stability and the short-term freeze stability.

## 4. Discussion

In the current study, the application of the relatively novel and “green” FPSE procedure for sample preparation, followed by LC–MS/MS analysis, provided a rapid, simple, and cost-effective approach, achieving high sensitivity and selectivity in the detection of ten common DOAs in OF and demonstrating promising performance for both screening and confirmatory analyses. The current work is an incremental extension of the previous brorphine paper [9], extending the FPSE-LC-MS/MS approach from a single analyte to a multi-analyte oral-fluid panel of common drugs of abuse and metabolites. A limitation of this study is the absence of authentic positive oral fluid samples, primarily due to practical and regulatory constraints, as oral fluid is not routinely collected or used for toxicological analysis in Greece. This restricts the evaluation of the method under real-case conditions.

The efficiency and selectivity of extraction in FPSE depend strongly on the choice of the fabric substrate and coating chemistry [8] in relation to the hydrophilicity/hydrophobicity of the target analytes. Cellulose and polyester substrates are used when solvent desorption is applied, whereas glass fibers are preferred for thermal desorption [18]. Due to the high polarity of most target analytes (having an n-octanol/water partition coefficient with logKow = 0.14–3.93 [19,20]), a polar polymer such as PEG coated onto a hydrophilic cellulose substrate was expected to exhibit improved elution efficiency. Furthermore, the porous sol–gel coating in combination with the high active surface area of the FPSE medium and the permeable cellulose substrate synergistically reduces the required time to reach extraction equilibrium. In addition, the low viscosity of OF allows rapid analyte diffusion [9]. The extended extraction time provided improved peak symmetry and reproducibility and was selected as optimal. The weak basic nature of most analytes (pKa = 8.18–9.9) [20,21], except for BE (pKa values 3.15 and 9.5 and an amphoteric behavior [22]), resulted in optimal results under neutral-pH conditions. Our results showed that most analytes exhibited higher elution efficiencies with methanol (polarity index 0.46 [23]).

The selected desorption conditions were optimal for eight out of ten analytes; methadone and methamphetamine behaved differently, possibly due to differences in polarity, size, and tertiary structure that affected the interactions with the PEG-based FPSE sorbent. Short desorption times resulted in incomplete analyte elution, whereas extended times did not further improve recovery and may have led to partial re-entrapment of the analytes in the FPSE medium. The selected low elution volume is in accordance with green chemistry practices [24,25]. Vortex-assisted desorption was preferred over ultrasound to avoid any possible alterations in the structural, physical, and chemical state of the substances that could be caused by thermal energy released in the ultrasonic bath.

Overall, the combination of FPSE’s equilibrium-based extraction, the analytes’ solubility and their ionization properties were the critical parameters affecting the recovery of analytes from OF, highlighting the significance of analyte–sorbent–solvent interactions in FPSE-based workflows. Specifically, the low recoveries could be the result of the limited water solubility of morphine [26,27] and BE (practically insoluble in water), while for EME, its low methanol solubility results in insufficient desorption from the FPSE membrane [28]. Nevertheless, the recoveries of all analytes were adequate to fulfill linearity, precision, and detectability criteria [29,30]. Although they had low recovery values, EME and morphine yielded acceptable analyte concentrations that allowed for detection at the lower levels recommended by international agencies for screening and confirmation purposes [31,32]. Finally, the use of fewer internal standards than the respective analytes can be acceptable to the forensic scientific community given that the presented methodology is adequately validated [30].

The current methodology for the determination of common DOA in OF demonstrates competitive sensitivity and offers notable advantages in simplicity and sustainability compared to other analytical protocols based on LC-MS/MS analysis with either conventional extraction techniques [1] or innovative and more elaborate approaches [12,13,14,15]. These LC–MS/MS approaches relied on extraction techniques such as QuEChERS salts (LOQs: 0.1–1.5 ng/mL) [12], microextraction by packed sorbent (LOQs: 0.5–5 ng/mL) [14], or dilute-and-shoot protocols (LOQs: 0.5–4.9 ng/mL) [1], achieving LOQs comparable to those obtained in the present study. However, they typically involved more elaborate sample preparation workflows. Moreover, a methodology using an elaborate liquid–liquid extraction with LC–MS/MS reported sub-ng/mL level LOQs (0.02–0.09 ng/mL) of analytes, albeit at the expense of increased solvent use, automation requirements, and procedural complexity [15].

On the other hand, FPSE is simple and cost-effective with low solvent consumption, meaning it can be characterized as a “green” methodology [6,7,8,9]. Nevertheless, its application in forensic toxicology is limited to selected novel psychoactive substances [9,10]. It is worth noting that the incorporation of the FPSE procedure prior to LC-MS/MS analysis for the sensitive detection of a multi-analyte OF panel of common DOAs and metabolites, as presented herein, broadens the potential application of FPSE in the field of forensic toxicology, adequately fulfilling the respective analytical requirements.

On the other hand, the GC–MS-based methodologies for DOA in OF generally present higher LOQs (2.9–20.6 ng/mL) than the current method, which may limit their applicability for low concentration or early detection scenarios [33]. It is worth mentioning that FPSE to OF analysis is reported in a limited number of studies, either on single compounds or restricted drug classes, such as novel psychoactive substances (brorphine [9] or fentanyl analogs [10]), rather than broad multi-analyte panels.

Last but not least is the alignment of the present method to international OF testing criteria from a regulatory and practical perspective; while it exceeds the performance of commonly used screening tools, the LOQs obtained for the tested analytes were substantially lower than the screening and confirmation cut-off concentrations recommended by international agencies [31,32].

Consequently, the proposed method highlights FPSE as a viable and sustainable alternative to conventional extraction techniques, fulfilling and substantially surpassing current regulatory sensitivity requirements by enabling the reliable detection of low-level drug exposure and recent drug use.

## 5. Conclusion

The proposed methodology combines high sensitivity and selectivity with simplified sample preparation and environmentally friendly operation, highlighting FPSE as a sustainable alternative to conventional extraction techniques that is fully compliant with international guidelines and demonstrates promising performance for clinical and forensic applications.

## Figures and Tables

**Table 1:** Analytical parameters selected for MRM: precursor (Q1) and product ions (Q3), retention times (RT), optimal declustering potential (DP), collision energy (CE), and collision cell exit potential (CXP). The entrance potential (EP) value for all analytes was 10. Qualifier ions are shown in bold under the Q3 column.

	Analytes	Q1 (*m*/*z*)	Q3 (*m*/*z*)	IS	DP (Volts)	CE (Volts)	CXP (Volts)
1	Amphetamine 1		**91**	IS1	25	25	12
	Amphetamine 2	136.0	119.1		25	13	20
	Amphetamine 3		65		25	51	10
2	BE 1		**168.2**	IS2	75	25	8
	BE 2	290.1	105		75	37	12
	BE 3		77		75	63	32
3	Cocaine 1		**182.2**	IS2	31	27	12
	Cocaine 2	304.0	82.1		31	41	12
	Cocaine 3		77		31	69	10
4	Codeine 1		**215.1**	IS1	100	35	14
	Codeine 2	300.1	165		100	51	18
	Codeine 3		183.1		100	39	16
5	EME 1		**182.1**	IS1	80	23	16
	EME 2	200.0	82		80	35	14
	EME 3		91.1		80	43	12
6	6-MAM 1		**165**	IS1	95	45	16
	6-MAM 2	328.0	211		95	37	16
	6-MAM 3		193.1		95	39	12
7	MDA 1		**163.1**	IS2	25	15	18
	MDA 2	180.0	105		25	31	12
	MDA 3		133.1		25	25	14
8	Methamphetamine 1		**91.1**	IS2	36	27	12
	Methamphetamine 2	150.1	119.2		36	15	10
	Methamphetamine 3		65.1		36	49	10
9	Methadone 1		**265.1**	IS2	20	21	16
	Methadone 2	310.1	105		20	33	12
	Methadone 3		77.1		20	67	12
10	Morphine 1		**201**	IS1	61	35	12
	Morphine 2	286.1	165		61	53	22
	Morphine 3		152		61	73	12
IS1	Morphine-d3 1	289.0	**155.1**		100	51	18
	Morphine-d3 2		165		100	49	20
	Morphine-d3 3		181		100	47	20
IS2	MDMA-d5 1		**165.1**		1	19	14
	MDMA-d5 2	199.1	107.1		1	35	12
	MDMA-d5 3		135.1		1	27	14

**Table 2:** Validation parameters of the presented method. Concentrations and LQC, MQC, and HQC are as listed in Section 2.9.2.

Analyte	LOD, ng/mL	LOQ, ng/mL	Linear Range, ng/mL	R^2^	Recovery % LQC MQC HQC			Matrix Effect % LQC HQC		Intraday Precision % LQC MQC HQC			Inter-Day Precision % LQC MQC HQC		
Amphetamine	0.4	1	1–60	0.992	64	64	64	−23	−22	9	8	7	12	11	10
BE	0.01	0.03	0.03–60	0.991	30	31	33	−9	−9	6	8	10	13	10	4
EME	1	4	4–60	0.991	10	11	11	−24	−20	4	5	3	8	6	3
Cocaine	0.01	0.03	0.03–60	0.991	71	65	70	−1	−7	8	7	6	4	5	4
Codeine	0.9	3	3–60	0.990	73	68	73	−2	−13	6	6	5	17	10	4
Methadone	0.01	0.03	0.03–60	0.999	93	92	87	7	8	3	5	6	7	8	11
Methamphetamine	0.4	1	1–60	0.994	60	59	61	−24	−24	2	3	3	6	7	6
Morphine	1	4	4–60	0.997	14	15	16	−10	−2	3	5	8	10	11	11
MDA	2	6	6–60	0.997	57	53	56	−11	−9	12	9	4	7	5	2
6-MAM	0.9	3	3–60	0.994	63	62	64	−24	24	2	2	2	6	6	7

## Data Availability

The original contributions presented in this study are included in the article/Supplementary Materials. Further inquiries can be directed to the corresponding author.

## Supplementary Materials

The following supporting information can be downloaded at: https://www.mdpi.com/article/10.3390/jox16030077/s1, Figure S1: Influence of coating material on the ten DOA yield; Figure S2: Influence of extraction on the ten DOA yield; Figure S3: Influence of pH on the ten DOA yield; Figure S4: Influence of solvent on the ten DOA yield; Figure S5: Influence of back-extraction time on the ten DOA yield; Figure S6: Influence of back extraction medium on the ten DOA yield.
