# Supplementary material for: Determination of Various Drugs of Abuse in Oral Fluid by a Fabric Phase Sorptive Extraction–LC-MS/MS Method
Source: J Xenobiot. 2026 May 3;16(3):77. doi: 10.3390/jox16030077 (PMC13214864; doi:10.3390/jox16030077)
Supplement: Supplementary file 1 [file jox-16-00077-s001.zip › jox-4256182-supplementary.pdf]

Journal of Xenobiotics

Supplementary Materials: Determination of Various Drugs of Abuse in Oral Fluid by a Fabric Phase Sorptive Extraction–LC-MS/MS Method

Dimitra Florou, Thalia Vlachou, Amvrosios Orfanidis, Vasilios Sakkas and Vassiliki A. Boumba

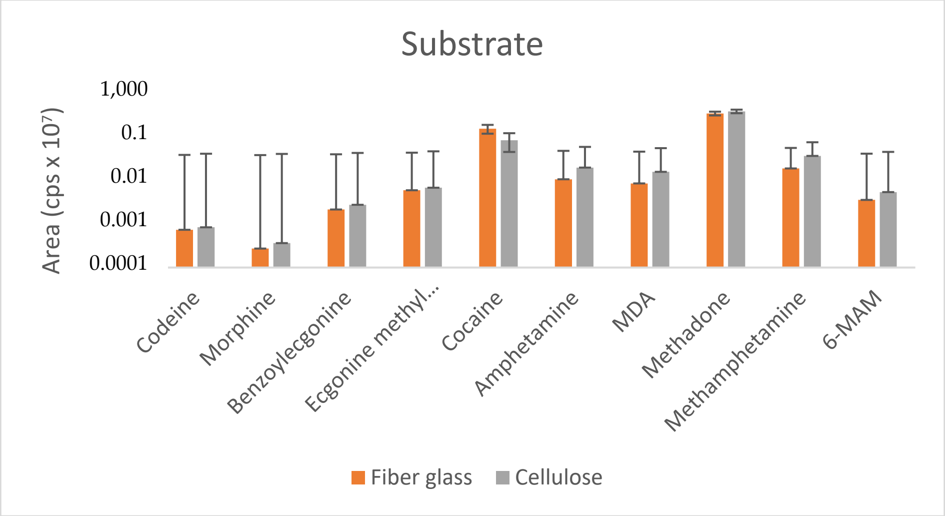

Figure S1. Influence of coating material on the ten DOA yield.

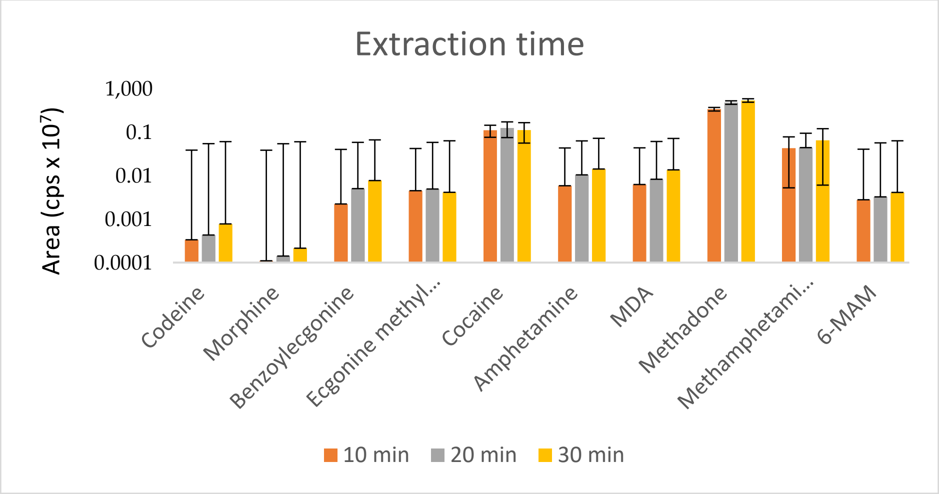

Figure S2. Influence of extraction on the ten DOA yield.

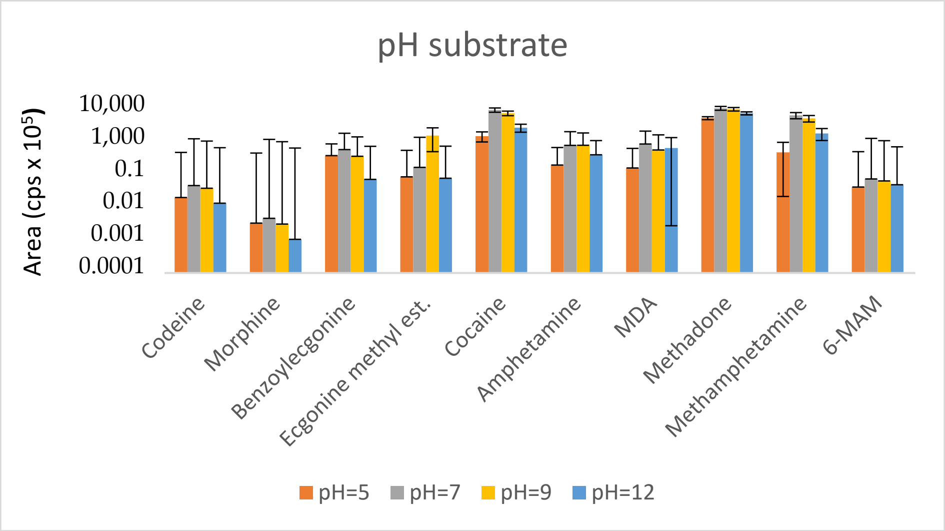

Figure S3. Influence of pH on the ten DOA yield.

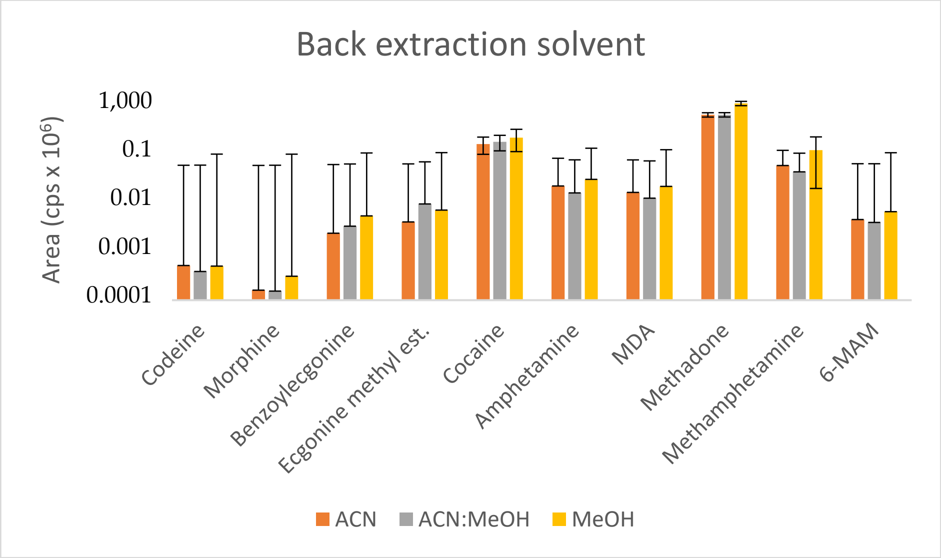

Figure S4. Influence of solvent on the ten DOA yield.

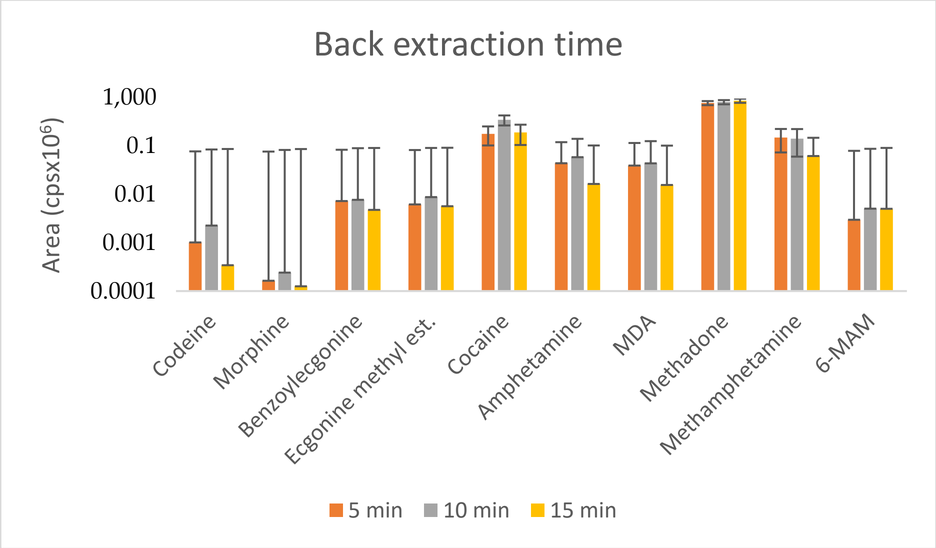

Figure S5. Influence of back-extraction time on the ten DOA yield.

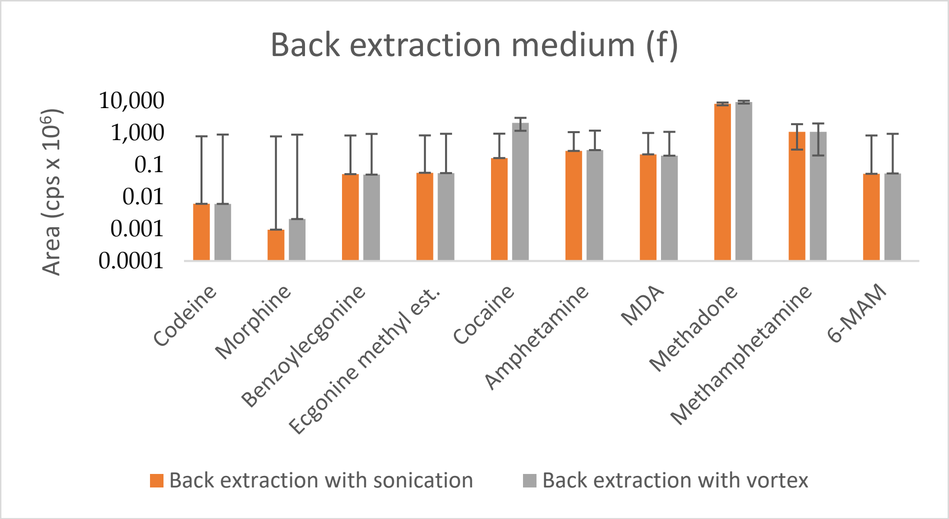

Figure S6. Influence of back extraction medium on the ten DOA yield.
